# Bacterial etiology of sputum from tuberculosis suspected patients and antibiogram of the isolates

**DOI:** 10.1186/s13104-020-05369-8

**Published:** 2020-11-10

**Authors:** Ramesh Sharma Regmi, Sujan Khadka, Sanjeep Sapkota, Sanjib Adhikari, Khuma Kumari Dhakal, Bishnu Dhakal, Bandana Lamsal, Sarad Chandra Kafle

**Affiliations:** 1 Department of Microbiology, Birendra Multiple Campus, Tribhuvan University, Bharatpur, Chitwan 44200 Nepal; 2 State Key Laboratory of Environmental Aquatic Chemistry, Research Center for Eco-Environmental Sciences, Chinese Academy of Sciences, Beijing, 100085 China; 3 State Key Laboratory of Respiratory Disease, Guangzhou Institutes of Biomedicine and Health, Chinese Academy of Sciences, Guangzhou, 510530 China; 4 University of Chinese Academy of Sciences, Beijing, 100049 China

**Keywords:** Tuberculosis, Respiratory tract infection, MDR, Sputum

## Abstract

**Objective:**

The current study aims to explore the bacteriology of sputum of tuberculosis (TB) suspected patients. A cross-sectional study was carried out in the sputum samples of 150 TB suspected patients visiting District Public Health Office, Bharatpur, Nepal. The samples were subjected to cultural, microscopic and biochemical analyses for the identification of the isolates. In addition, antibiotic susceptibility tests were carried out with a special focus on ESBL and MBL production following Clinical and Laboratory Standard Institute guidelines.

**Results:**

Bacterial growth was recovered in 47% (71/150) of the TB suspected patients of which 12.66% (19/150) had pulmonary TB infection. *Streptococcus* spp. (9%) and *Pseudomonas aeruginosa* (9%) were the most frequently isolated bacteria. Enterobacteriaceae accounted for 35% of the total isolates. Occurrence of bacterial pathogens was more in males (69%) than in females (31%).The incidence of bacterial pathogen was seen associated with gender of the patients and with the TB infection (p < 0.05) but independent with age of the patients and HIV infection (p > 0.05). Tetracycline was effective against *Streptococcus* spp. whereas gentamicin was effective against *Bacillus* species. Imipenem and co-trimoxazole were effective drugs for Gram-negative isolates. Among 83 isolates, 35 were multi-drug resistant, 9 were ESBL producers and 4 were MBL producers.

## Introduction

Respiratory Tract Infections (RTIs) are the most frequently reported among human infections, out of which Lower Respiratory Tract Infections (LRTIs) account for almost 90% [1]. Upper Respiratory Tract Infections (URTIs) are commonly caused by viruses than bacteria and fungi but LRTIs are more commonly caused by bacteria and less by fungi and viruses. These diseases directly result in about 7 million deaths annually [2]. The HIV pandemic has even worsened morbidity and mortality due to LRTIs which causes about 70% of illnesses in AIDS patients [3]. To differentiate Tuberculosis (TB) from other LRTIs such as bacterial pneumonia is an important clinical challenge in developing countries, and failure to differentiate TB from other LRTIs may result in poorer health outcomes which may lead in high mortality rate [4]. Tuberculosis is the most feared health issue in developing countries like Nepal. Emergence of bacterial coinfections along with the development of antimicrobial resistance complicates the TB-treatment process [5].

Several studies carried out world-wide report that the potent pathogens of RTIs are *Streptococcus pneumoniae*, *Haemophilus influenzae*, *Klebsiella pneumoniae*, *Pseudomonas aeruginosa*, *Escherichia coli*, *Staphylococcus aureus*, *Bacillus* spp., *Moraxella catarrhalis*, *Streptococcus pyogenes* and some other enteric Gram-negative rods such as *Salmonella choleraesuis*, *Citrobacter koseri* [6, 7]. Most of these bacteria are normal flora of the human respiratory tract. So it is clear that most of the time, the infection is initiated by normal flora and secondary infection from other invader bacteria [8].

Gram-negative bacteria, especially Enterobacteriaceae are increasing their antibiotic resistance ability [9]. Gram-positive cocci such as *S. aureus and Streptococcus* spp. are also evolving as multi-drug resistant (MDR) [10]. To the best of our knowledge, no any attempts have been made yet exploring the bacterial composition of sputum of TB suspected patients in Nepal. Hence, this study was designed to explore the bacteriological spectrum of sputum from the patients suspected of tuberculosis and determine the antibiotic susceptibility pattern of the isolates.

## Main text

### Study design, area and sample population

This was a descriptive cross-sectional study enrolling 150 TB suspected patients who visited District Public Health Laboratory (DPHO), Bharatpur, Chitwan over 2 months from January to February, 2020. The patients were asked to collect early morning sputum sample in a clean dry and leak-proof container and bring it cautiously to DPHO laboratory.

### Sample collection and transport

Immediately after collecting the sputum, each sample was observed microscopically for the presence of AFB bacilli by Ziehl–Neelsen staining at DPHO laboratory. The samples were transported aseptically to the Microbiology laboratory of Birendra Multiple Campus for bacteriological investigations. A little of each AFB positive sample was left at the DPHO laboratory for further analysis by Genexpert method.

### Culture and identification of the isolates

The methodology was followed according to a similar study done by Ngekeng [6]. A loopful of the uncentrifuged sample was streaked over Chocolate agar, Blood agar, MacConkey agar and XLD agar (Hi-Media, India) under laminar airflow conditions. Blood agar was incubated at anaerobic condition and Chocolate agar, MacConkey agar and XLD agar were incubated at the aerobic condition for 24 h at 37 °C. Identification of bacterial isolates was done based on their morphological and biochemical characteristics [11].

### Antibiotic susceptibility testing

Antibiotic Susceptibility Test was done by modified Kirby Bauer's disc diffusion method following CLSI guidelines (2016) [12]. Altogether, 17 different commonly used antibiotics procured from Hi-Media, India were used for testing. In case of *Bacillus* spp., AST was performed as suggested by Charteris et al. [13].

### Screening of ESBL and MBL producers

Primary screening of ESBL producers was done by using ceftazidime (CAZ) (30 µg) and cefotaxime (CTX) (30 µg) disks (Hi-Media, India). If the zone of inhibition was 22 mm for CAZ and/or 27 mm for CTX, the isolate was considered a potential ESBL-producer as recommended by NCCLS [14]. Combination disk method [15] was used to confirm ESBL-producing isolates in which CTX and CAZ (30 µg), alone and in combination with clavulanic acid (CA) (10 µg) were used. An increase in ZOI of 5 mm for either antimicrobial agent tested in combination with CA versus its zone when tested alone confirmed ESBL [12]. Imipenem resistant Gram-negative isolates were selected for the further detection of MBL production by disc potentiation method using imipenem (10 µg) and meropenem (10 µg) with and without EDTA (1 µg) as previously described [24].

### Quality control

Each batch of media and reagents was subjected to sterility and performance testing. During antibiotic susceptibility test, quality control was done using the control strains of *E. coli* ATCC 25922.

### Data management and statistical analysis

All the raw data obtained in this study were tabulated in SPSS V.20 and Chi-square test was performed. P ≤ 0.05 was assigned as significant.

## Results

### Distribution of bacteria in sputum

**Fig. 1:**
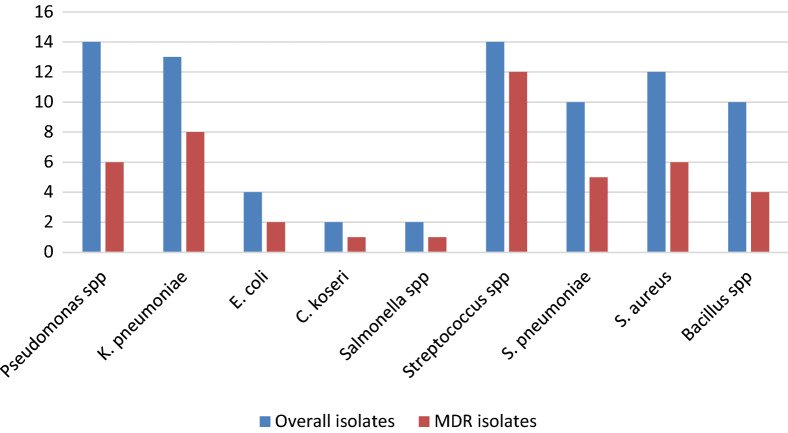
Distribution of MDR and overall bacterial isolates

Among the 150 sputum samples, only 71 (47.33%) showed the bacterial growth, out of which 61 (85.92%) had monomicrobial growth and 10 (14.09%) had polymicrobial growth. The most common bacteria were *Streptococcus* spp. and *Pseudomonas aeruginosa*, both isolated in 14(9.33%) samples. Enterobacteriaceae isolates were obtained in 21 instances, among which *K. pneumoniae* was predominant 13 (8.67%) (Fig. 1).

### Association of different variables with bacterial isolation

Among 150 study participants, only 5 (3.33%) people were HIV-infected of which 4 (80.00%) showed the bacterial growth. The incidence of bacterial growth was not found associated with HIV infection (p > 0.05). Gender-wise, 54.44% of growth was recovered from males and 36.67% from females and recovery of bacteria in sputum sample was found associated with gender (p < 0.05). Higher prevalence of LRTIs was found in the age group 31–45 and 46–60 accounting for 14 (56.00%) and 29 (55.76%) of growth respectively. However, no statistical association was observed between bacterial incidence with the age group (p > 0.05). Among 150 TB suspected patients, only 12.67% were confirmed to have tuberculosis of which 18 (94.74%) showed the growth of bacteria. A very high degree of association was noted between the incidence of bacterial pathogens and tuberculosis infection (p < 0.05). Current smokers were found to be more vulnerable to bacterial infection as compared to non-smokers and past smokers as 65.79% isolates were recovered from the current smokers. A strong association was observed statistically between the smoking habit and bacterial growth (p < 0.05). Habit of alcohol consumption was also noted to contribute significantly in bacterial infection as 78.57% alcohol consumers showed growth of bacteria (p < 0.05) (Table1).

**Table 1:** Distribution of bacterial growth with various attributes

Attributes	Sample size	Growth rate (%)	Odds ratio (95% CI)	p-value
Gender				
Male	90	54.44	2.06 (1.06–4.03)	0.033
Female	60	36.66	1	
Age group				
0–15	9	4.44	1	
16–30	29	37.93	0.76 (0.17–3.47)	0.727
31–45	25	56.00	1.59 (0.34–7.37)	0.553
46–60	52	55.76	1.58 (0.38–6.54)	0.531
60+	35	37.14	0.74 (0.17–3.25)	0.689
Smoking				
Yes	38	65.79	2.65 (1.16–6.04)	0.020
No	69	42.03	1	
In past	43	39.53	0.9 (0.42–1.96)	0.794
Alcohol consumption				
Yes	28	78.57	5.84 (2.14–15.97)	0.000
No	83	38.55	1	
In past	39	43.59	1.23 (0.57–2.67)	0.597
TB infection				
Yes	19	94.74	26.49 (3.43–204.46)	0.001
No	131	40.46	1	
HIV infection				
Yes	5	80.00	4.66(0.51–42.68)	0.174
No	145	46.21	1	

### Antibiotic susceptibility pattern of the isolates

Antibiogram of the *Streptococcus* spp. showed tetracycline was most effective drug with 86.33% sensitivity, whereas gentamicin was least effective with 14.30% sensitivity. All Enterobacteriaceae isolates were resistant to amoxicillin/clavulanic acid whereas sensitive to co-trimoxazole. *P. aeruginosa* was sensitive to some of the second-line antibiotics whereas amikacin was completely resisted by it (Additional file 1: Tables S1 and S2). Among total 81 bacterial isolates, 45 were multi-drug resistant (MDR). *Streptococcus* spp. was predominant (26.67%) among the MDR isolates (Fig. 1). To confirm ESBL producers, 14 presumed ESBL producing isolates were tested among which 9 (25.71%) isolates were confirmed ESBL producers, *K. pneumoniae* 4 (44.44%) being the predominant one. On performing combined disk test of 10 presumptive MBL isolates, only 4 (40.00%) were confirmed as MBL producers of which 2 isolates were *P. aeruginosa* and 1 each of *K. pneumoniae* and *E. coli* (Table 2).

**Table 2:** Frequency of ESBL and MBL isolates

Organisms	ESBL		MBL	
	Presumptive	Confirmatory (combination disk method)	Presumptive	Confirmatory (combined disk test)
*P. aeruginosa*	4	2 (50.00%)	5	2 (40.00%)
*K. pneumoniae*	7	4 (57.14%)	3	1 (33.33%)
*E. coli*	2	2 (100.00%)	2	1 (50.00%)
*Salmonella* spp*.*	1	1 (100.00%)	0	0
Total	14	9 (64.29%)	10	4 (40.00%)

## Discussion

This study documented bacterial growth in the sputum of 47.33% (71/150) tuberculosis-suspected individuals. A Cambodian study also revealed similar result with 43.79% bacterial growth [4]. On the other hand, report from a Nigerian study showed a growth rate of around 61.37% [6]. Such a high degree of variation may be due to the different methods employed for the collection and processing of the samples in different places. In the current study, *P. aeruginosa* was the most predominant bacterial isolate recovered in 9.33% of the total sample. A similar result was found in a research done in Pakistan, where the prevalence of *P. aeruginosa* was11.96% [16]. The frequent incidence of *P. aeruginosa* in the sputum may be owing to their ability to colonize a wide range of ecological niches, such as air polluting agents, animal hosts and humans [17]. *S. pneumoniae* was isolated in 6.67% sputum samples in our study, but in most of the other similar world-wide studies, *S. pneumoniae* has been reported as the chief isolate [6, 18, 19]. In the present study, the prevalence of *K. pneumoniae* was 8.67% in the sputum samples. This rate is almost similar to the study conducted by Hasan et al. who observed a 10.8% prevalence of *K. pneumoniae* [20]. Prevalence of *Staphylococcus aureus* was 8.00% in this study, similar to Ngekeng’s study where it was 6.5% [6]. Incidence of *Streptococcus* spp., *S. aureus* and *K. pneumoniae* may be due to their distribution in the respiratory tract where they remain as opportunistic pathogens and may infect when patient’s immune system is compromised. We observed a prevalence of 6.67% for *Bacillus* spp. which is exactly similar to the study carried out in Iraq [20].

Prevalence of LRTIs was found greater in male (54.44%) than in female (36.67%). A similar study in Nigeria showed LRTIs was 54.80% in male and 67.10% in female [6]. Gender has varied impact on bacterial incidence as some studies show higher infection in females [19] while others show more in males [18]. Higher growth reported among men in our study might be due to lifestyle factors like smoking and alcohol consumption [6]. Higher prevalence of infection was seen in the age group 31–45 (56.00%) and 46–60 (55.76%). A similar study by Attia et al. showed the age group 38–65 (53.00%) as the most susceptible for LRTIs [4]. Age group of 46–60 are under more threats of being infected as people in this age group have declined level of immunity and their exposure to the environment is high [21]. In our study, 12.67% of the suspected patients were confirmed to have TB. This rate is similar to the study carried by Ngekeng et al. where it was 13.79% [6]. Tuberculosis infection was significantly associated with bacterial co-infection (p < 0.05). As immunity decreases during active TB, bacteria attack the immune-compromised person more easily as compared to healthier person [22]. In the current study, only 5 (3.33%) people were HIV-infected patients and no any association was observed between having HIV with bacterial incidence (p > 0.05). This study accounted 65.79% bacterial growth from the smokers. This rate is higher than the study carried out by Ngekeng where it was 61.10% [6]. In a study, Attia and his colleagues found the staggering rate of 81.81% where a significant association between smoking and bacterial growth was not noted [4]. The rate of bacterial growth among alcohol consumers was 78.57% in this study, which is 53.9% in a study carried out in Nigeria [6]. Higher prevalence of bacterial growth in those who were habitual to smoking and alcoholism may be because smoking and alcoholism usually increase risk to lower respiratory tract infections by diminishing mucosal immunity [18].

The present study documented 55.56% MDR isolates which is higher than a study carried out in Kathmandu (47.57%) [23]. Another study in the same city also showed similar prevalence of MDR isolates (57.50%) [24]. Besides, 26.00% of Gram-negative isolates were ESBL producers in this study, *K. pneumoniae* being the top contributor. This rate is similar to the one carried out by Pokhrel and his colleagues where the rate was 24.27% with the same organism being dominant. This study also documented 11.43% of Gram-negative isolates as MBL producers. A study carried out in LRTIs patients in Nepal Medical College revealed 5.80% isolates were MBL producers [24]. Another study in Nigeria accounted 4.70% Gram-negative isolates as MBL producer [25].

## Conclusions

The current study reveals a bacterial prevalence of 47.33% from the sputum of TB-suspected patients. Out of 150 study subjects, 12.66% (19/150) had pulmonary tuberculosis. Discovery of multidrug resistant bacteria including EBL and MBL producers in the sputum of patients with TB and LRTIs is worrisome and concerned authorities should be more alert to abate their incidence and dissemination.

## Limitations

The present study doesn’t indicate whether the TB suspected patients had some other LRTIs such as pneumonia. Also, it does not include other important LRTIs causing bacteria such as *H. influenzae* and *M. catarrhalis.*

## Supplementary information

**Additional file 1: Table S1.** Antibiotic susceptibility pattern of Gram-positive isolates. **Table S2.** Antibiotic susceptibilty pattern of Gram-negative isolates.

## Data Availability

All the data obtained during this study are available within the article.

## Abbreviations

TB: Tuberculosis
ESBL: Extended spectrum beta-lactamase
MBL: Metallo beta-lactamase
MDR: Multidrug resistant
AST: Antibiotic susceptibility test
CLSI: Clinical and laboratory standards institute
ZOI: Zone of inhibition
