# Supplementary material for: Bacterial etiology of sputum from tuberculosis suspected patients and antibiogram of the isolates
Source: BMC Res Notes. 2020 Nov 10;13:520. doi: 10.1186/s13104-020-05369-8 (PMC7654065; doi:10.1186/s13104-020-05369-8)
Supplement: Supplementary file 1 — Additional file 1: Table S1. Antibiotic susceptibility pattern of Gram-positive isolates. Table S2. Antibiotic susceptibilty pattern of Gram-negative isolates. [file 13104_2020_5369_MOESM1_ESM.docx]

**Table S1:** Antibiotic resistance pattern of Gram-positives isolates

| ANTIBIOTICS | *Streptococcus* spp. | | | | | S. *pneumoniae* | | | S. *aureus* | | | *Bacillus* spp*.* | | | |
| --- | --- | --- | --- | --- | --- | --- | --- | --- | --- | --- | --- | --- | --- | --- | --- |
|  | S | I | | R | | S | I | R | S | I | R | S | | I | R |
| Vancomycin (30µg) | 21% | 7% | | 72% | | 18% | 0% | 82% | 75% | 17% | 8% | 27% | | 27% | 46% |
| Gentamicin (10µg) | 14% | 7% | | 79% | | 46% | 18% | 36% | 66% | 17% | 17% | 100% | | 0% | 0% |
| Chloramphenicol (30µg) | 43% | 21% | | 36% | | 0% | 46% | 54% | 84% | 8% | 8% | 82% | | 18% | 0% |
| Levofloxacin (5 µg) | 64% | 21% | | 15% | | 36% | 28% | 36% | 33% | 25% | 42% | 18% | | 0% | 82% |
| Erythromycin (15µg) | 14% | 50% | | 36% | | 64% | 18% | 18% | 33% | 8% | 59% | 36% | | 18% | 46% |
| Cefepime (30µg) | 43% | 14% | | 43% | | 54% | 28% | 18% | NT | | | 27% | | 36% | 36% |
| Penicillin (10 units) | 71% | 7% | | 22% | | NT | | | NT | | | 54% | | 0% | 36% |
| Ceftazidime (30µg) | NT | | | | | NT | | | NT | | | 46% | 0% | | 54% |
| Tetracycline (30µg) | 86% | | 0% | | 14% | 82% | 0% | 18% | 84% | 8% | 8% |  | |  |  |
| Amoxicillin/Clavulinic Acid (20/10µg) | NT | | | | | 82% | 9% | 9% | NT | | | NT | | | |
| Oxacillin (1 µg) | NT | | | | | NT | | | 42% | 16% | 42% | NT | | | |
| Co-Trimoxazole (25µg) | NT | | | | | NT | | | 92% | 8% | 0% | NT | | | |

S= Sensitive, R= Resistant, I= Intermediate, NT= Not tested

**Table S2**: Antibiotic resistance pattern of Gram-negative isolates

| Antibiotics | *P. aeruginosa* | | | Enterobacteriaceae | | |
| --- | --- | --- | --- | --- | --- | --- |
|  | S | I | R | S | I | R |
| Gentamicin(10µg) | 100.00% | 0% | 0% | NT | | |
| Polymixin B(30µg) | 63.45% | 14.15% | 21.40% | NT | | |
| Ceftazidime(30µg) | 78.56% | 21.44% | 0% | 52.38% | 9.52% | 38.10% |
| Ciprofloxacin(5µg) | 78.56% | 7.22% | 14.22% | 91.10% | 4.45% | 4.45% |
| Amikacin(30µg) | 0% | 0% | 100.00% | 91.10% | 8.90% | 0% |
| Imipenem(10µg) | 64.29% | 0% | 35.71% | 71.43% | 0% | 28.57% |
| Amoxicillin/Clavulinic Acid(20/10µg) | 92.50% | 0% | 7.50% | 24.67% | 29.33% | 47.00% |
| Tetracycline(30µg) | NT | | | 48.50% | 38.35% | 13.15% |
| Aztreonam (30 µg) | NT | | | 51.10% | 29.45% | 29.45% |
| Co-trimoxazole(25µg) | 21.25% | 21.25% | 57.50% | 100.00% | 0% | 0% |

S= Sensitive, R= Resistant, I= Intermediate, NT= Not tested
